# Full-Thickness Skin Graft from the Submental Triangle for Facial Defect Reconstruction

**DOI:** 10.3390/jcm12093195

**Published:** 2023-04-29

**Authors:** Chih-Chun Hou, Yu-Yu Chou, Shyi-Gen Chen, Chih-Hsin Wang, Yuan-Sheng Tzeng

**Affiliations:** Division of Plastic Surgery, Department of Surgery, Tri-Service General Hospital, National Defense Medical Center, Taipei 114, Taiwan

**Keywords:** cutaneous malignancies, donor site, esthetic outcomes, non-melanocytic tumors

## Abstract

Full-thickness skin grafting plays an important role in facial reconstruction for excessive skin defects or possible eye, nose, or lip distortion after a malignant tumor excision. For esthetic consideration, full-thickness skin grafts of the retroauricular region can be used, but the amount of skin is limited. The skin of the submental triangle has similar cutaneous features and provides a large graft. We aimed to evaluate the feasibility of using full-thickness skin grafts harvested from the submental triangle to reconstruct facial skin defects. We retrospectively analyzed 14 cases treated at a single institution to evaluate the clinical and esthetic outcomes, and patients’ satisfaction. During 6–24 months of follow-up, the wounds healed with well-matched color, texture, and contour. No obvious hypertrophies or distortions were observed. Additionally, the removal of redundant submental triangle skin resulted in the secondary gain of double chin reduction. All patients were satisfied with the appearance of both donor and recipient sites. In conclusion, the submental triangle is a good possible option for full-thickness skin grafts used to resurface facial skin defects. Using this approach, both donor and recipient sites can achieve satisfactory esthetic outcomes.

## 1. Introduction

Surgical-wide excision is a gold standard for treating malignant skin tumors [1]. Following excision, different options are available for managing skin defects depending on the site, size, and type of tissue lining. Primary closure is usually the first choice for small lesions. However, direct closure is not possible owing to excessive skin defects; it may lead to distortion of the eyes, nose, or lips. As part of the reconstruction ladder, skin grafts and flaps offer an alternative for surgeons to address this condition.

When treating facial skin defects, esthetics should be considered. In general, flaps are transferred from an area near the defect, which can provide a similar skin color and texture [2]. Compared to skin grafts, flaps lengthen the skin incision; sometimes, the surgical wounds do not lie along the Langer’s lines of the face, which can result in unpleasant surgical scars. In the reconstruction of facial skin defects, full-thickness skin grafts can provide a minimal secondary graft contraction and fine skin quality [3], which also results in good cosmetic outcomes.

The most common full-thickness skin graft donor sites include the retroauricular region, supraclavicular region, inguinal fold, buttock fold, hypothenar area, and anterior wrist fold [4]. For esthetic considerations, a match in color, texture, and adnexal quality is required. A full-thickness skin graft from the retroauricular region can be used, but only a limited amount of skin is available. The skin of the submental triangle has very similar cutaneous features. Furthermore, age-related laxity and skin redundancy result in larger amounts of harvestable skin, and the secondary gain of a double chin contouring effect [5].

In this study, we used a full-thickness skin graft harvested from the submental triangle to reconstruct facial skin defects and aimed to identify the clinical and esthetic outcomes.

## 2. Materials and Methods

### 2.1. Study Design

This is a single institutional, retrospective, observational case series study. We included all patients who underwent facial skin reconstruction using full-thickness skin grafts harvested from the submental triangle to resurface facial skin defects caused by the excision of facial skin tumors, between January 2019 and January 2021. Demographic data, including sex, age, body mass index (BMI), pathology of the skin tumor, location and dimensions of the skin defect, dimensions of the skin graft, preoperative and postoperative photographs, and medical comorbidities, were collected.

Fourteen patients were included in the study. We used the Manchester Scar Scale to evaluate postoperative esthetic outcomes [6]. The Manchester Scar Scale is a visual analog scale consisting of five different aspects: color, finish, contour, distortion, and texture. The score ranged from 5 to 18, with a lower score representing a better esthetic outcome. The patients’ overall outcome satisfaction was self-reported using a scale of satisfaction ranging from 1 to 10 (1–2: not at all satisfied, 3–4: slightly satisfied, 5–6: neither satisfied nor dissatisfied, 7–8: very satisfied, 9–10: extremely satisfied). Both the Manchester Scar Scale and the scale of satisfaction level were evaluated every 3 months after the operation, until 12 months after the operation. For cases with a follow-up period of only 6 months, we used the assessment at the 6th month, and for cases with a follow-up period of more than 12 months, we uniformly used the assessment at the 12th month.

Informed consent for the surgical procedures and photography was obtained from each patient, and the study was approved by the Institutional Review Board of Tri-Service General Hospital (approval number C202005135, dated 26 August 2021). This study was conducted in accordance with the principles of the Declaration of Helsinki.

### 2.2. Surgical Technique

**Figure 1:**
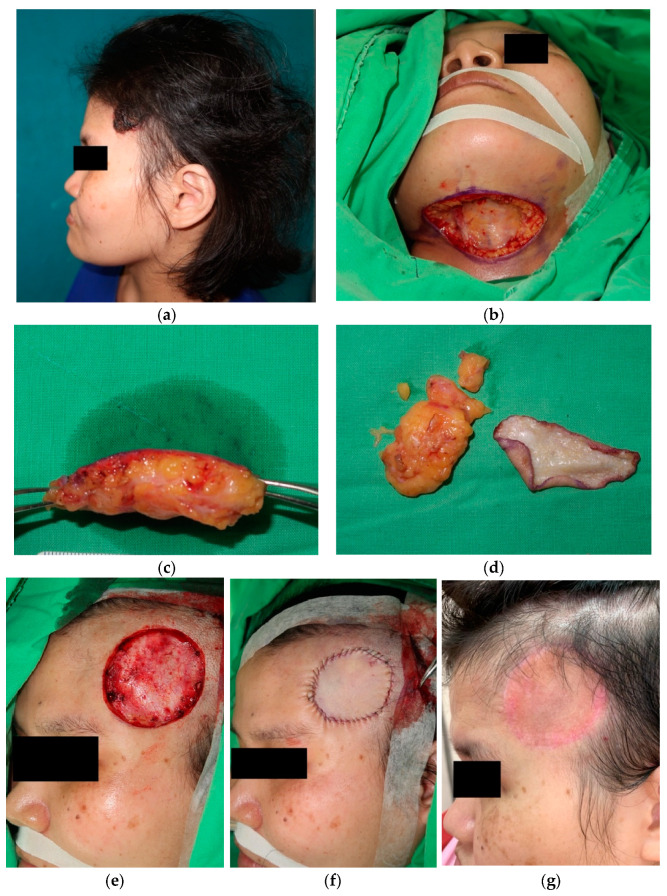
Case 3: (**a**) A 43-year-old woman with basal cell carcinoma (BCC) on her left temple region that measured 4.5 × 4.0 cm^2^ in size. (**b**,**c**) Harvesting full-thickness skin graft from the submental region, (**d**) de-fatting procedure, (**e**) skin defect after wide excision of BCC measuring 6.5 × 5.0 cm^2^, (**f**) appearance immediately after operation, and (**g**) follow-up photo at 3 months.

Following the excision of the facial skin lesions, we measured the size of the skin defects. In order to avoid the deformity of the donor site, we marked a larger skin area over the submental triangle. All patients demonstrated skin redundancy and laxity in the submental triangle. Full-thickness skin grafts were harvested from the donor site, and the underlying soft tissue and fat were trimmed off (Figure 1c,d). The skin grafts were sutured using 6-0 nylon and tied over a bolster of Vaseline-impregnated gauze and fluffed gauze. According to the situation of each case, we made opportune adjustments, including undermining the wound ends and lengthening the wound. The donor site was closed in layers using a 4-0 vicryl suture for the subcutaneous layer and 5-0 nylon for the skin. We also performed liposuction once the dog’s ear was prominent, or a fatty neck presented.

## 3. Results

**Figure 2:**
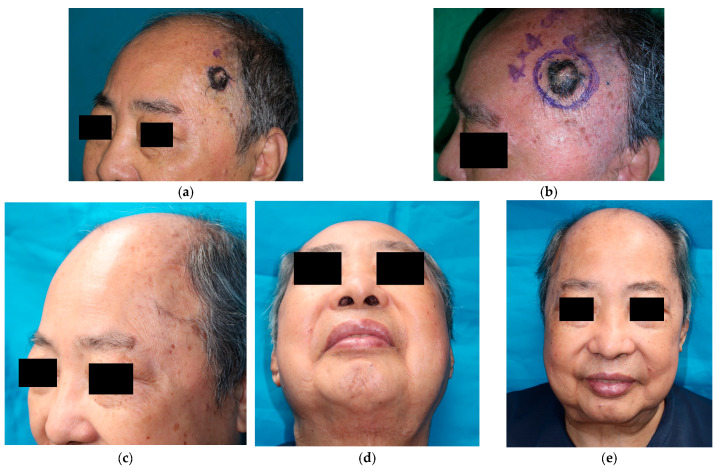
Case 7: A 75-year-old man with a pre-cancerous skin lesion on the left temple, 2.5 cm in diameter. (**a**) Preoperative appearance. (**b**) The skin defect is 4.0 × 4.0 cm^2^ in size. (**c**–**e**) Appearance at the 2-year follow-up.

**Figure 3:**
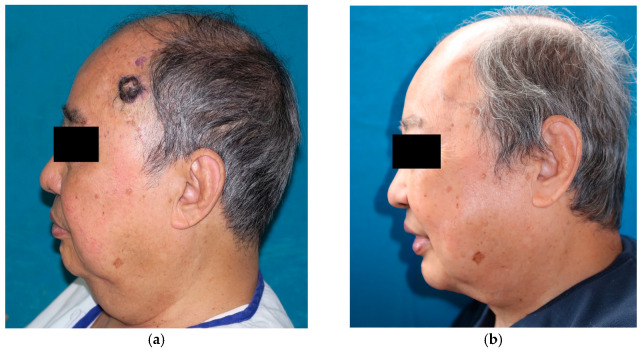
Case 7 (continued): Comparison of neckline: (**a**) The preoperative appearance and (**b**) appearance at the 2-year follow-up. An esthetically pleasing and youthful jawline is achieved as secondary gain.

The study included seven women and seven men aged between 43 and 91 years, 70.6 years in average. Most cases (10 patients) were of basal cell carcinoma. There were other pre-cancerous skin tumors in three patients, and squamous cell carcinoma in one patient. The dimensions of the skin defects averaged 2.88 × 2.39 cm^2^ (ranging from 1.5 × 1.0 cm^2^ to 4.5 × 4.0 cm^2^). The frozen section of the excised skin lesion showed free margins in all cases, and full-thickness skin grafts were harvested from the submental triangle. The dimensions of the skin graft harvested from the submental triangle averaged 4.54 × 3.07 cm^2^ (ranged from 3.0 × 1.5 cm^2^ to 6.5 × 5.0 cm^2^). Details are presented in Table 1. The patients were discharged 1–2 days after the operation. No immediate complications were noted. The tie-over dressing was removed 5–7 days after the operation, and the dressing was changed in the outpatient department. No graft failure or poor healing at the donor site was observed. All patients were followed up for 6–24 months. There is no cancer recurrence or distant metastasis. The Manchester Scar Scale scores averaged 8.57 (ranged from 6 to 11). No obvious hypertrophies or distortions were observed. The surgical scar at the submental triangle was hidden below the chin and was not clearly visible from the front (Figure 2e). The removal of redundant skin, which shaped the neck contour, resulted in effects similar to those of a neck lift (Figure 3b). An esthetically pleasing and youthful jawline was achieved as a secondary gain. All patients were satisfied with the appearance of their neck jawlines. Patients’ overall outcome satisfaction averaged 8.85 (a range from 7 to 10) on a scale of satisfaction level.

Medical comorbidities and lifestyle habits are listed in Table 2. The most common comorbidity in our study was hypertension, and half of the patients in our study presented with diabetes and smoking habits. One of the patients had a history of psoriasis requiring topical steroids. Two patients had undergone previous chemotherapy, one for rectal cancer and one for leukemia, with the rectal cancer patient also receiving radiation therapy. No patient had a history of previous head and neck radiation exposure.

Case 3: A 43-year-old woman had a poorly healed, crusted skin lesion over her left temporal region for 3–4 years (Figure 1a). She visited a local dermatology clinic where a chronic wound with secondary infection was initially suspected. Fusidic acid cream had been applied for 2 months without an improvement of the lesion. Due to a high suspicion of malignancy, she was referred to our department for excision and pathological examination, which revealed basal cell carcinoma with an elevated, dome-shaped, easily bleeding, golden brownish crust with peripheral erythema. The skin tumor lesion was excised with a 5 mm safe margin and deep into the fascia, which proved to be free of tumor invasion in the frozen section. The skin defect over the left temporal region was 4.5 × 4.0 cm^2^ in size (Figure 1e), and a full-thickness skin graft measuring 6.5 × 5.0 cm^2^ was harvested from submental triangle (Figure 1b).

At the 3-month follow-up, the skin graft was well matched in texture and contour (Figure 1g). Only a pale pink color around the previous suture sites was noted. Decreased skin redundancy over the submental triangle was noted without donor site morbidity.

Case 7: A 75-year-old man had a progressively enlarging skin lesion over his left temporal scalp for several years (Figure 2a). The examination revealed that he had brownish and blackish hyperpigmented skin lesions measuring 2.5 × 2.0 cm^2^ with a central ulcer approximately 0.7 × 0.5 cm^2^ in size, deep into the subcutaneous layer. The skin lesions were excised with a free margin of frozen section. The pathological examination revealed seborrheic keratosis with severe dysplasia. The skin defect over the left temporal scalp was 4.0 × 4.0 cm^2^ in size (Figure 2b), and a full-thickness skin graft measuring 6.0 × 4.5 cm^2^ was harvested from the submental triangle.

At the 2-year follow-up, the skin graft had healed well with perfect matching of skin color, texture, and contour (Figure 2c). There were no complications, and the donor site recovered with minimal scar formation (Figure 2d), which was not conspicuous when viewed from the front (Figure 2e).

Before the operation, the patient presented with a poor neckline and an obvious double chin (Figure 3a). At postoperative year 2, the cervicomental angle and neck contour were improved. The neckline was evident, presenting with an effect similar to that of a neck lift (Figure 3b).

## 4. Discussion

The management of patients with facial skin cancer is challenging, and esthetic outcomes should always be considered. The treatment options include surgical excision, cryotherapy, electrodesiccation, and curettage. Surgical excision is the standard treatment owing to a lower risk of recurrence than with other techniques [1,7]. Unfortunately, all available surgeries leave a scar. Thus, the goal of reconstruction of facial skin defects should target the restoration of the patients’ usual appearance.

We have different reconstruction options for managing skin defects in the reconstruction ladder. It is important to assess the characteristics of facial skin defects, including the size, location, and type of tissue lining, before choosing a suitable approach [8]. Direct closure is the simplest and fastest method. However, it is not suitable for larger skin defects. In some cases, although direct closure can be performed, it may lead to a distortion of the eyes, nose, or lips. Thus, skin grafts and flaps should be considered when dealing with such conditions [9].

Skin grafts are classified as split-thickness and full-thickness skin grafts. Both types of skin grafts may result in primary and secondary contractures [10]. In secondary contractures, the split-thickness skin graft deforms the appearance and results in a less esthetic match. Thus, full-thickness skin grafts are preferable for the reconstruction of skin defects in facial regions, where cosmetic outcomes play an important role.

Common local flaps include advancement, transposition, and rotation flaps, which provide similar skin color and texture [11]. For larger defects, regional flaps such as nasolabial or forehead flaps are used.

From a subjective point of view, Ebrahimi et al. compared the results between the local flap and the skin graft for the reconstruction of a cheek skin defect [12]. There were 40 patients with cheek skin defects who were treated with local flaps (20 patients) and skin grafts (20 patients). The results showed that there was no difference in satisfaction and tissue coordination at the 12-month follow-up, but the skin color had improved in the local flap group. Since only 12 among the 20 patients in the skin graft group were treated with full-thickness skin grafts, and the remaining 8 were treated with split-thickness skin grafts, the ratio of similarity in skin color at the follow-up was significantly lower when compared with the local flap group.

From an objective point of view, Martinovic et al. assessed the skin quality in the facial region following reconstruction with local flaps and full-thickness skin grafts [13]. There were 31 patients treated with local flaps and 30 patients treated with full-thickness skin grafts. When comparing the aspect of hydration level and transepidermal water loss in the two groups, the local flap group resulted in better outcomes than the full-thickness skin graft group because the local flap consisted of full-thickness skin with subcutaneous tissue [13]. In the mechanism of the wound-healing process, the local flap undoubtedly has better skin quality; unfortunately, the evaluation of the patients’ satisfaction and esthetics was not included in the study.

While flaps theoretically result in better cosmetic effects than skin grafts, they also require longer skin incisions. In many cases, surgical wounds do not lie along the Langer’s lines of the face, which may result in noticeable scars [2]. Considering regional differences, Asian patients tend to experience hyperpigmentation and a high risk of worse scarring after injury [14]. In our experience, most of our patients hesitated to lengthen the surgical incision, especially in the facial region. Therefore, if skin defects do not present with exposed cartilage, exposed bone, or underlying neurovascular structures, reconstruction with flaps is not always necessary.

Regarding postoperative complications, Mamsen et al. discussed the differences between local flaps and full-thickness skin grafts in the reconstruction of facial defects [15]. There was a total of 607 patients with facial skin defects who were treated with local flaps (304 patients) and skin grafts (303 patients). When evaluating infection and wound dehiscence, there was no significant difference between the two groups, but regarding hematoma and necrosis, they were significantly worse in the full-thickness skin graft group, possibly because they included larger skin defects for reconstruction in the full-thickness skin graft group [15]. Although the study stated that there was no significant difference in the surgical experience between the two groups, we believe that attentive hemostasis during the operation and the stable fixation of skin grafts can greatly reduce the complications of hematoma and necrosis. Hence, there were no complications in our cases.

In our study, all patients received full-thickness skin grafts for the reconstruction of facial skin defects. Considering the complexion, texture, and photo-damage, full-thickness skin grafts harvested from the retroauricular region and the frontal region at the hair border appear to be the best candidates. Unfortunately, the retroauricular region can only provide a limited amount of skin, and it can be challenging when confronted with larger skin defects. The frontal region is also an effective and fine donor site choice because the scar can be hidden at the hair border. However, if the facial skin defect is large, it may not be possible to provide a sufficiently sized and intact skin graft. On the other hand, care should be taken if we choose the frontal region at the hair border as the donor site choice for a full-thickness skin graft for male patients. Because there is a greater possibility of alopecia in males, unpleasant surgical scars might become visible. In case 3, the area of the skin defect over the left temporal region was 4.5 × 4.0 cm^2^ (Figure 1e), which made harvesting a full-thickness skin graft from the retroauricular region or the frontal region impossible. However, in older people, sagging and loose skin of the submental triangle can usually be found, and it presents with a similar complexion and texture to those of the facial region, making it a possible alternative (Figure 1g). This way, the scar of the donor site is not conspicuous when viewed from the front, and the wound will not extend onto the face. Nevertheless, some medical conditions will affect the skin quality of the submental triangle, such as previous head and neck radiation exposure, previous neck trauma, and previous surgery in the neck region, which may also impair wound healing of the donor site. Especially in patients who underwent previous head and neck radiation exposure or previous neck lymph nodes dissection, lymphedema might occur as complication of the donor site. Awareness should be given to the above situations. Other reconstruction methods are recommended.

There are only a few studies on the application of full-thickness skin grafts harvested from the submental triangle. Its first clinical use was reported in 2002 by Field et al. [16]. A full-thickness skin graft was applied to a skin defect over the nose. However, long-term outcomes were not documented. Artsi et al. harvested full-thickness skin grafts from the submental triangle and anterior neck to cover a skin defect of the periocular region [5]. In their study, five patients underwent an excision of the skin lesions over the periocular regions and immediate reconstruction using full-thickness skin grafts. All the patients presented with good cosmetic outcomes. Eroğlu et al. applied full-thickness skin grafts to different parts of the face, including the nasal tip, nasal dorsum, nasal sidewall, forehead, temporal region, cheek, and chin [17]. In their study, a slightly decreased pigmentation was observed, but an excellent match in texture and thickness was noted in the long-term follow-up. In addition to the advantage of resemblance in cutaneous features, the skin laxity of the submental and neck regions provides sufficient volume for coverage of many types of skin defects. Hanna et al. reported on the application of a full-thickness skin graft harvested from the submental and neck regions to cover the donor site of a radial forearm free flap [18]. This resulted in favorable esthetic and functional outcomes of the affected wrist.

Owing to the thick subcutaneous fat in the submental triangle, it is relatively safe to harvest skin from this region. The donor site is primarily closed after a full-thickness skin graft is harvested from the submental triangle, as the redundant skin is removed. It presents effects similar to those of a neck lift. In case 7, the cervicomental angle improved immediately after the operation and at the 2-year follow-up (Figure 3b). Care should be taken to avoid a simple elliptical incision yielding the deformity of the donor site. Moreover, excessive tension at the midline of the donor site could create a bow-string deformity in the neck. Therefore, we made opportune adjustments, such as liposuction, undermining the wound ends, and lengthening the wound, according to each case’s particular situation. Although there was a surgical scar at the submental triangle, it was hidden below the chin and was not clearly visible from the front (Figure 2e). All patients were satisfied with the appearance of their donor sites. Additionally, the skin of the submental triangle was well matched in color, texture, and contour of the facial region in all cases. Therefore, both donor and recipient sites achieved satisfactory esthetic outcomes, which added to patient satisfaction.

Possible complications of the recipient site include infection, hematoma, wound dehiscence, and graft necrosis, which make careful hemostasis and rigid fixation of skin grafts essential. On the other hand, wound dehiscence, seroma, hematoma, and fat necrosis might happen as complications of the donor site, so attentive hemostasis and meticulous suture with layers by layers during operation is very important. Luckily, infection and unacceptable scars of the donor site rarely occur due to the easy maintenance of skin cleanliness of the neck and skin laxity of the submental region. Although the number of cases enrolled in our study was small and there was a relatively high proportion of comorbidities that could impair wound healing, no complications occurred at either the donor or recipient sites in our study.

Based on the results of our study, we believe that full-thickness skin grafts harvested from the submental triangle are a good option for the reconstruction of facial skin defects. The limitations of our study include the small sample size and a lack of long-term follow-ups of outcomes and recurrence. Furthermore, this is a single institutional, retrospective, case series study without a comparison group, such as a reconstruction with full-thickness skin grafts harvested from other donor sites, or with local flaps, which increases the odds of bias. Future prospective trials, with a larger sample size and a long-term follow-up of at least a year, are needed to validate these results. Recurrence also needs to be assessed in future studies.

In conclusion, the skin of the submental triangle provides a good possible option for good full-thickness skin grafts, especially for the reconstruction of skin defects in the facial region. It is particularly useful in elderly patients who have redundant neck skin or double chins. Using this method, both donor and recipient sites can achieve satisfactory esthetic outcomes.

## Figures and Tables

**Table 1:** Details of 14 patients included in this study who received full-thickness skin grafts from the submental triangle.

Case	Sex	Age (Years)	Pathology	Location	Dimensions of Facial Skin Defect (cm^2^)	Dimensions of Skin Graft Harvested from Submental Triangle (cm^2^)	Manchester Scar Scale Score	Scale of Satisfaction Level
1	F	73	BCC	Nasal sidewall	2.2 × 1.7	4.0 × 2.5	8	10
2	M	57	BCC	Left eminence of concha	2.0 × 2.0	4.0 × 2.5	9	10
3	F	43	BCC	Left temporal region	4.5 × 4.0	6.5 × 5.0	9	9
4	M	90	BCC	Left buccal region	3.5 × 2.8	5.0 × 3.5	9	9
5	M	56	BCC	Right temporal region	4.2 × 3.5	6.0 × 4.0	11	7
6	F	60	BCC	Nasal tip	2.0 × 2.0	4.0 × 2.5	8	8
7	M	75	Seborrheic keratosis with severe dysplasia	Left temporal region	4.0 × 4.0	6.0 × 4.5	6	10
8	F	90	BCC	Nasal bridge	1.7 × 1.5	2.5 × 2.0	7	10
9	M	67	SCC in situ	Left buccal region	2.5 × 1.5	4.0 × 2.0	9	8
10	M	58	BCC	Right buccal region	3.0 × 2.0	5.0 × 2.5	8	9
11	M	75	BCC	Left nasal alar region	1.5 × 1.0	3.0 × 1.5	8	8
12	F	84	Microcytic adnexal carcinoma	Right nasal alar region	2.2 × 1.7	4.0 × 2.0	10	8
13	F	91	BCC	Left temporal region	3.0 × 2.7	3.5 × 3.5	9	9
14	F	70	Seborrheic keratosis with severe dysplasia	Left postauricular region	4.0 × 3.0	6.0 × 5.0	9	9

Abbreviations: F, female; M, male; BCC, basal cell carcinoma; SCC, squamous cell carcinoma.

**Table 2:** Details of medical comorbidities and lifestyle habits in this study.

Medical Comorbidities and Lifestyle Habits	Patients Number
Hypertension	8
Heart disease	6
Diabetes	7
Hypothyroidism	1
Autoimmune disease	1
Obesity (BMI > 30)	1
Smoking	7
Alcoholism	1
Previous radiation therapy	1
Previous chemotherapy	2

## Data Availability

The data presented in this study are openly available.
